# Qualitative assessment of climate-driven ecological shifts in the Caspian Sea

**DOI:** 10.1371/journal.pone.0176892

**Published:** 2017-05-05

**Authors:** Omid Beyraghdar Kashkooli, Joachim Gröger, Ismael Núñez-Riboni

**Affiliations:** 1 Thünen Institute of Sea Fisheries, Hamburg, Germany; 2 Institute for Biosciences, University of Rostock, Rostock, Germany; 3 Department of Natural Resources, Isfahan University of Technology, Isfahan, Iran; Universidade de Aveiro, PORTUGAL

## Abstract

The worldwide occurrence of complex climate-induced ecological shifts in marine systems is one of the major challenges in sustainable bio-resources management. The occurrence of ecological environment-driven shifts was studied in the Southern Caspian Sea using the “shiftogram” method on available fisheries-related (i.e. commercially important bentho-pelagic fish stocks) ecological and climatic variables. As indicators of potential environmentally driven shift patterns we used indices for the North Atlantic Oscillation, the Southern Oscillation, the Siberian High, the East Atlantic-West Russia pattern, as well as Sea Surface Temperature and surface chlorophyll-a concentration. Given the explorative findings from the serial shift analyses, the cascading and serial order of multiple shift events in climatic-ecologic conditions of the southern Caspian Sea suggested a linkage between external forces and dynamics of ecosystem components and structures in the following order: global-scale climate forces lead to local environmental processes, which in turn lead to biological components dynamics. For the first time, this study indicates that ecological shifts are an integral component of bentho-pelagic subsystem regulatory processes and dynamics. Qualitative correspondence of biological responses of bentho-pelagic stocks to climatic events is one of the supporting evidences that overall Caspian ecosystem structures and functioning might have–at least partially–been impacted by global-scale climatic or local environmental shifts. These findings may help to foster a regional Ecosystem-based Approach to Management (EAM) as an integral part of bentho-pelagic fisheries management plans.

## Introduction

Perceiving how ecosystem components and interactions are impacted by anthropogenic activities (e.g. fishing) as well as considering the surrounding environment is required for a reasonable ecosystem-based management [1]. The principle is that—apart from the influences of exploitation on fish populations—the main mechanisms controlling fish population dynamics are alterations in ecosystem forces, such as “climate–ocean impacts and trophic level interactions” [2]. Global fisheries processes and activities are inevitably under climate variability regimes [3] and the fisheries productivity, fish population dispersal and variability, intensively rely on the environment status [4]and climate dynamics [5]. Climate forces are exogenous, superintendent and latent drivers which induce profound large-scale alterations in marine ecosystems by impacting their environmental state. Environmental variability is a critical and significant component for marine ecosystem production and recovery capacity [6].

Identifying, characterizing and evaluating complex behavior of ecosystem components in response to external physical drivers–particularly those related to ongoing environmental changes–is considered to be fundamental towards fostering an Ecosystem-based Approach to Management (EAM) to sustainably exploit living resources. Debates and discourses about climate change and sustainability of marine resources are rising [7] and there are plenty of evidences which indicate the effects of climate variations on various ecosystems [5]. Climate change is impacting many natural populations such as fish and therefore marine and estuarine fisheries [8, 9]. For management purposes, perception of nature, extent and spatial distribution of present and upcoming climate change effects is necessary [9].

When looking for complex climate-driven impacts in marine resources, two major modalities can be recognized. On the one hand, marine population dynamics may be affected by gradual trends, continuous cycles and temporal variability of climate forcing factors (e.g. [10–13]. On the other hand, sudden and high amplitude climatic perturbations may induce dramatic alterations in marine ecological regimes. Generally, the first type of impacts can be investigated by quantitative correlational analysis of climate-biological datasets [14]. However, the intricate reactions of ecosystem components due to abrupt climate alterations (which is known as climate-induced “regime shifts”) are qualitatively studied by comparing successive ecological states. Regime shift is being accounted as a global issue in aquatic ecosystems context with widely-documented and still growing evidences in several marine systems [11, 15–23]. By combining various sets of aspects, [24] suggested a comprehensive, multi-criterial and “standard” definition for marine ecosystem regime shifts involving the occurrence of sudden, high-amplitude and low-frequency changes over a large geographical area that are perceptible in several features of the ecological components (biotic and abiotic).

Climate-induced regime shifts have profound implications for sustainability of marine resources and fisheries production [20, 21, 23, 24]. The worldwide occurrence of complex climate-induced ecological shifts in marine systems (which has been documented substantially) is one of the major challenges in bio-resources management [22–27]. Identifying potential shift patterns (as well as relevant causes) in ecosystem components, structures and processes might lead to important implications for ecosystem-centered strategies aiming to achieve a sustainable long-term fisheries governance agenda.

During recent decades the unique ecosystem and biodiversity of the Caspian Sea has been under enormous pressure of numerous anthropogenic stressors and is encountering a multitude of ecological challenges such as industrial and biological contamination [28–31], sea level fluctuation [32–34], fisheries overexploitation, management failure and collapse of commercial fish stocks [35–37], illegal fishing and poaching [35, 38, 39], invasion of *Mnemiopsis leidyi* [39–42], eutrophication [43, 44], loss of biodiversity [45, 46], environmental mismanagement [47, 48], etc. On top of these pressures, the Caspian Sea environment and bio-resources have been—and will be—inevitably exposed to on-going global climatic changes (like other marine ecosystems); nevertheless, climate is still a largely-ignored issue in marine resources management in this region.

Some previous studies found that the thermal conditions and sea level in the Caspian Sea were associated to large scale climatic phenomenon such as the Southern Oscillation (SO) [32, 49], the North Atlantic Oscillation (NAO) [32, 50] and the Siberian High (SH) system [49, 51]. The proposed mechanism is generally related to the role of global atmospheric circulation forces on regulating moisture and heat fluxes over the Caspian region. However, most of those studies (based on correlation analysis) did not consider the possibility that the relation between large-scale climate variables and local conditions of the Caspian Sea could be through triggering of regime shifts. Moreover, a further influence of such climatic or local environmental changes in the Caspian Sea and local fisheries has been, so far, not yet documented. In the present study, we try to fulfill these gaps with a statistical method that identifies various types of regime changes.

Considering the importance of ecological shifts for fisheries sustainability in the context of EAM, this research for the first time attempted to provide a qualitative perspective on the occurrence of potential ecological climate-driven shifts in the Caspian Sea. This study was mainly focused on the regional investigation of potentially-visible shift patterns, dynamics and causalities by applying a qualitative-statistical approach called “shiftogram” [20]on a range of fisheries-related ecological proxies and climatic variables in the southern Caspian Sea. The fundamental working hypothesis was that o*bvious shifts in ecological proxies are in compliance with environmental factors shifts*.

## Material and methodology

### Study area and fish species

**Fig 1:**
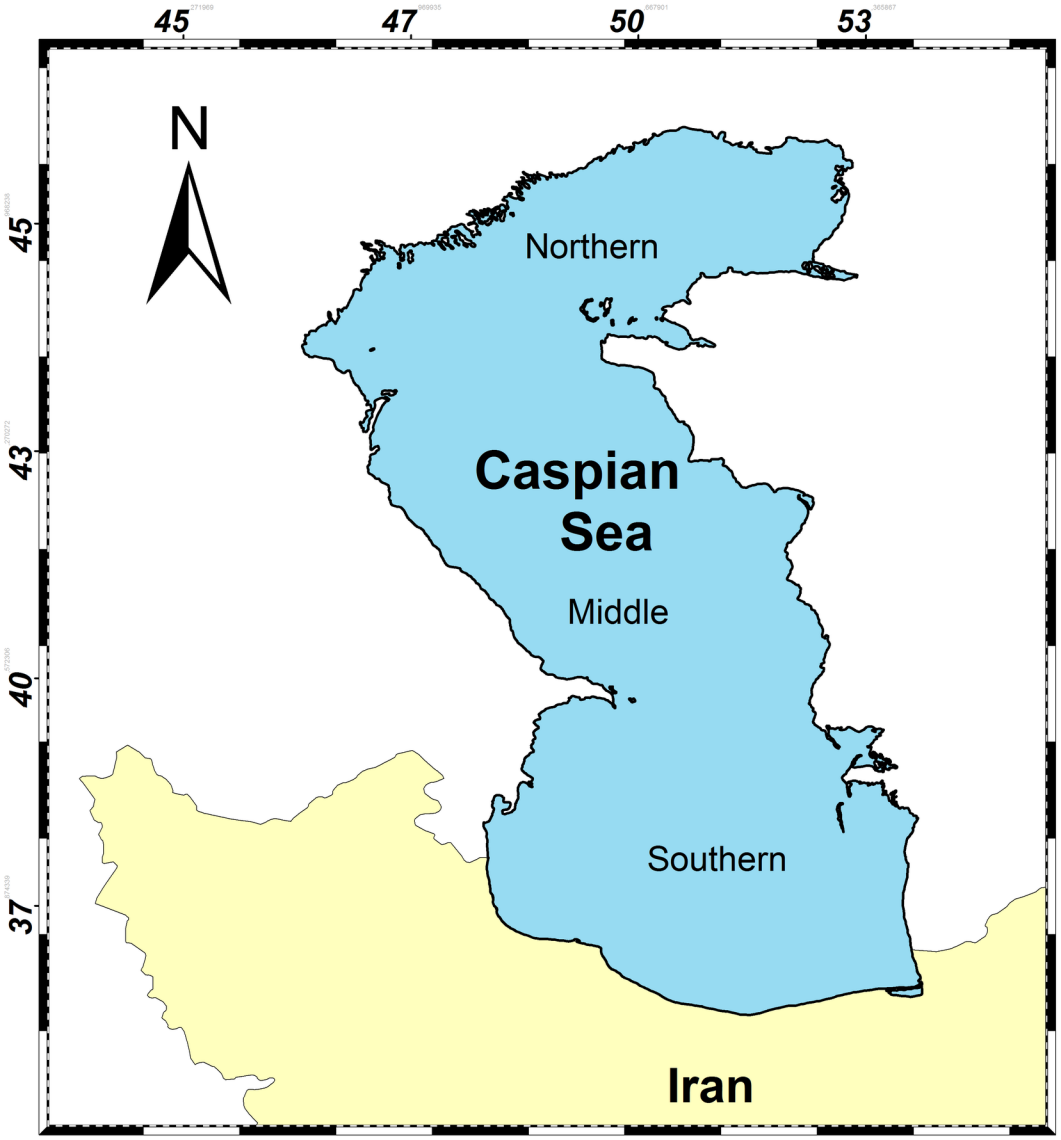
Map of the Caspian Sea.

The Caspian Sea, the largest enclosed water body of the earth, is located between Asia and Europe. The Caspian Sea is generally divided into three parts (considerably differing in physico-geographical features) namely, the northern, middle and southern part. Our study focuses on the deeper southern part(Fig 1).

Among 115 fish species and sub-species inhabiting this ecosystem, demersal Chondrichthyes sturgeons, and bentho-pelagic bony fish including kutum, mullets, breams, carps, barbus, salmons, as well as pelagic kilka species have been historically important in commercial fisheries of the Caspian Sea [46].

Exploitation of bony fish (started since 1927) in Iranian waters of the Caspian Sea has always played a prominent role in fisheries economy of the southern region [46, 52, 53]. The target bentho-pelagic species Caspian kutum (*Rutilus frissi kutum* (Kamensky, 1901)) and the golden grey mullet, (*Liza aurata* (Risso, 1810)) constitute the major commercial fisheries catches in Iranian coastal waters of the Caspian Sea [46, 53–55].

The Caspian kutum (*Rutilus frisii kutum* (Kamensky, 1901)), is a medium size endemic fish with a life span of 9–10 years in the southern Caspian Sea. Due to the nutritional value and palatable taste for consumers, as well as its profitability for fishermen, kutum is considered as the most important bony fish of the Caspian sea [46], covering more than 70% of the annual Iranian costal catches [56]. After kutum, *L*. *aurata* is the second most dominant fish in commercial catch composition of bony fish in the southern Caspian Sea coastal zones [57]. Also with regards to the increasing market demands, it is considered as one of the most valuable economic resources for local fishermen [57]. With regards to the legal restrictions for commercial catch of sturgeon species (due to the risk of extinction) during the last two decades [58–61] as well as the recent collapse of kilka stocks in the Caspian Sea [35–37], both kutum and mullet are currently considered as the main target species for the entire fisheries in the southern coasts.

### Data description

The datasets used in this study belong to three categories: regional fisheries-derived ecological datasets, large-scale climatic indices and local satellite-based environmental time series. Their properties will be described in the subsequent sections, while their time scopes and data providers are shown in Table 1.

**Table 1:** Summary of the data series used in this study.

*Name*	*Period*	*Origin*
***Regional fisheries-derived data***		
The Caspian kutum stock assessment data	1991–2011	From IFRO reports, [63]; Hasan Fazli, personal communication
The golden grey mullet stock assessment data	1991–2011	From IFRO reports, [63]; Hasan Fazli, personal communication
***Large-scale climatic indices***		
North Atlantic Oscillation (NAO)	1899–2011	NCAR/UCAR
Southern Oscillation (SO)	1951–2011	CPC/NCEP/NOAA
Siberian High (SH)	1900–2011	[71]; NCEP/NCAR/NOAA Earth System Research Laboratory; Ismael Núñez-Riboni
East Atlantic-West Russian (EA-WR)	1950–2011	CPC/NCEP/NOAA
***Local satellite-based environmental data***		
Sea Surface Temperature (SST)	1982–2011	NOAA Earth System Research Laboratory; Ismael Núñez-Riboni
Upper-layer Chlorophyll-a concentration (Chl-a)	1998–2011	ACRI-ST GlobColour service supported by EU FP7 MyOcean and SA GlobColour Projects; Boris Cisewski, personal communication

#### Regional fisheries-derived ecological data

Multi-aspect regional data-provision constraints and improprieties in less studied areas such as the southern Caspian Sea always impose many limitations in long-term ecological dynamics research (particularly in climate-driven ecological dynamics studies). Hence, only the long-term, regular and reliable fisheries-based time series of two major commercial bentho-pelagic species were used: the Caspian Kutum and the golden grey mullet.

For both species, the most important population components (to serve as ecological proxies) including spawning (parental) stock biomass (SSB) and the recruit (R) numbers (complete series over a period of 1991–2011) were collected. The relevant data series were obtained and compiled from the final reports of the Iranian Fisheries Research Organization (IFRO) on the stock assessment of bony fishes (based on the biomass cohort analysis method, [62]) in Iranian waters of the Caspian Sea [63]. Recruitment numbers were estimated by dividing the biomass of the youngest fish observed in the catch composition (in both species age 2) by the average individual weight of this age (calculated from von Bertalanffy length-weight equation for each year; based on the IFRO reports). SSB estimates (metric tons) were extracted from the biomass through multiplying the biomass by the maturity index (based on the IFRO reports; Dr. Hasan Fazli, personal communication) for each age. Logarithmic-transformed data series were then used for the shift analysis.

#### Large-scale climatic indices

Large-scale climatic indices are diagnostic quantities reflecting major modes of climatic variability. The following global climate indices were selected and examined as potential indicators of climatic fluctuations and variability (i.e. capture at least a fraction of variability) over the study area: North Atlantic Oscillation, the Southern Oscillation, East Atlantic-West Russia pattern and Siberian High.

The North Atlantic Oscillation (NAO) is one important global atmospheric variability pattern with inter-annual to inter-decadal fluctuations, exhibiting a wide range of impacts on ecological systems dynamics and processes [64, 65]. The NAO strongly influences the atmospheric circulations and weather conditions, especially during the winter in the northern hemisphere [66]. In this study, we use an NAO index based on principal component analysis (PCA) obtained from the NCAR/UCAR (U.S. National Center for Atmospheric Research/University Corporation for Atmospheric Research) website (https://www.climatedataguide.ucar.edu/climate-data/hurrell-north-atlantic-oscillation-nao-index-pc-based). The annual average index was utilized as first broad-scale climate proxy to investigate the potential climate-driven ecological shifts.

The Southern Oscillation (SO) is the major inter-annual climate variability mode of the world. The Southern Oscillation index (SOI) represents the strength of the so called El-Niño and La-Niña phases of the Southern Oscillation [67–69]. Annual averages of SOI were obtained for the period 1951-2011from the CPC/NCEP/NOAA (Climate Prediction Center / National Centers for Environmental Prediction / U.S. National Oceanic and Atmospheric Administration) website (http://www.cpc.ncep.noaa.gov/data/indices/soi).

In the northern hemisphere, the Siberian High (SH) is considered one of the main air pressure centers and atmospheric-circulation systems during winter [70]. We computed the SHI from sea level pressure data from the NCEP/NCAR (U.S. National Centers for Environmental Prediction /the National Center for Atmospheric Research) reanalysis from the NOAA Earth System Research Laboratory website (http://www.esrl.noaa.gov/psd/data/gridded/data.ncep.reanalysis.derived.surface.html)based on the analysis of [71]. By comparing our index with the one calculated by [71](which is based on the climatology of [72]) for the period of 1900–2001, an offset of 0.6 was added to our index so that both indices matched for the years 1996–2009.

The East Atlantic-West Russia (EA-WR) pattern is a teleconnection pattern affecting the climatic state and weather condition of the Eurasian region and consists of two major pressure/height anomaly cores centered on the Caspian Sea and western regions of Europe [73, 74]. We used annual averages of the EA-WR index obtained from the CPC/NCEP/NOAA website (http://www.cpc.ncep.noaa.gov/data/teledoc/eawruss.shtml).

#### Local satellite-based environmental data

Aside from the global-scale climatic indices, we were also interested on investigating the potential impacts of local-scale hydrographic variables on the species dynamics. Therefore, we used satellite-derived Sea Surface Temperature (SST) and surface chlorophyll-a concentration (Chl-a) to represent potential local hydrographic variations in our analyses. SST is one of the most crucial and straightforward-in-touch ambient properties for all aquatic organisms. The upper-layer chlorophyll-a concentration can reflect the potential basal-trophic productivity in marine ecological webs which could be assumed to be sequentially transitioned through the ecological chain influencing higher-trophic level organisms–such as fish.

We use the weekly averaged NOAA Earth System Research Laboratory’s SST with resolution of 1°×1° from the NOAA website (http://www.esrl.noaa.gov/psd/data/gridded/data.noaa.oisst.v2.highres.html). We calculated annual averages of the southern part of the Caspian Sea (south of 40° N) from 1982 to 2011.

Surface chlorophyll-a concentration, as a qualitative indicator of phytoplankton biomass, was obtained from the global satellite products of ACRI-ST GlobColour service supported by EU FP7 MyOcean and SA GlobColour Projects (downloaded from http://hermes.acri.fr). Annual averages of the surface chlorophyll-a concentrations for the Southern Caspian Sea (spatially-enclosed to 50° to 52°E and 38° to 40°N) were calculated (from 1998 to 2011).

### Methodology

In this study we adopt an algorithm for the identification of shiftsin time series developed by [20], known as “shiftogram” approach being inspired from studying structural breaks in econometric time series models. Compared to other shift detection methods, this approach offers several technical preferences and some benefits: powerful hypothetical context, objective and flexible selection/localization of different break types, accurate and multi-criteria statistical diagnostics testing, dealing with rather short time series and with robust manifold properties. It has also been applied successfully in several studies as an explorative “screening device” to characterize the types and locations of potential shifts in some marine studies, in both cases of univariate and complex multivariate set-ups, as well as in different levels of ecological organization and time scales [11, 19, 20, 75].

This approach is based on modeling structural breaks in time series by inserting external shift (indicator) variables as additional predictors in a linear regression model for a given response variable y_t_ over time. A pulse ${\text{D}}_{\text{t}}^{\text{P}}$ (at t_0_) and a step ${\text{D}}_{\text{t}}^{\text{S}}$ at (t_0_+1) intervention variable are appended to a basic time regression model (deterministic time trend model) for each input variable:

$${\text{y}}_{\text{t}}=\beta_{0}+\beta_{1}\text{t}+\alpha_{1}{\text{D}}_{\text{t}}^{\text{P}}+\alpha_{2}{\text{D}}_{\text{t}}^{\text{S}}+\varepsilon_{\text{t}}$$ (1)

Where the α’s and β’s are regression parameters while ε_t_ is a white noise error term. ${\text{D}}_{\text{t}}^{\text{P}}$ and ${\text{D}}_{\text{t}}^{\text{S}}$ are defined as:

$${\text{D}}_{\text{t}}^{\text{S}}=\{\begin{array}{ll} 1 & \text{if t}={\text{t}}_{0}\\ 0 & \text{otherwise} \end{array}\;"\text{Pulse intervention}"$$

and

$${\text{D}}_{\text{t}}^{\text{P}}=\{\begin{array}{ll} 1 & \text{if }{\text{t}>\text{t}}_{0}\\ 0 & \text{otherwise} \end{array}\;"\text{Step intervention}"$$

A potential breakpoint at t_0_ iteratively passes along a given time series by consecutively incrementing t_0_ by one year each time step. In each iteration, Ordinary Least Square (OLS) is used to solve the extended regression equation. Pertinent statistical proof criteria, like model-fit-measure and a series of marginal p-values, are recorded during each iteration. In order to simplify the investigation and interpretation of shift analysis, the original data series and the iterations outputs are represented graphically in a set of a 10-plots template (entitled as “shiftogram”) comprising the following elements presented in Table 2:

**Table 2:** A set of 10-plots comprising a “shiftogram”.

*Plot*	*Shiftogram Elements*
**1**	Original time series to be tested
**2**	Bias corrected Akaike’s information criterion (AICc; [76]) as a measure of quality-of-fit
**3**	p-value regarding the F-test of joint significance of all parameters in the break model
**4**	Illustrates the statistical power (1-β) measures as an indication for the chance of incorrect no-warning (β = type II error); by increasing the power the risk of incorrect no-warning decreases
**5**	1storder autocorrelation coefficient AR (1)of the break-model residuals
**6**	p-value related to AR (1)
**7**	p-value of the F-test of the pure impulse (a shock)
**8**	p-value of the F-test for detecting a change in the slope
**9**	p-value of an ANOVA F-test on the similarity of the pre- and post-break levels
**10**	p-value of a Levene‘s test on the homogeneity of the pre- and post-break variances

While the procedures behind panel plots 1 to 7 use the entire time series, for the procedures behind panel plots 8 to 10 a symmetric window before and after t_0_may be specified to reflect the “small-scale behavior” of a time series in the vicinity of t_0_ [20]. For specifying this window, a rule of thumb is using 20% of the length of time series (by considering the sample size and potential cyclic element in the time series [20]).

According to [11] plots 2 (AICc), 3 (p-joint) and 4 (power) are the major reference indicators for recognizing the locality of the shift (temporal positioning), while all other plots are informative about the changes in properties of a time series and are beneficial for distinguishing the shift type. The shiftogram panels 2, 3 and 4 (i.e. AICc, p-joint statistics and power surrounded by red rectangles) are considered as the key-statistical indicators for recognizing the year of the shift.

The occurrence of climate-induced shifts was evaluated by comparing the timing of potential shifts in each ecological and climatic series. Since an occurrence of climate shifts in the past may also influence the species, the shift inspection process for the climatic time series was initialized 4 years (20% length of time series) before the beginning of the species population time series.

All data analysis has been performed here using SAS 9.4 and the “shiftogram” module (SAS macro, by Prof. Dr. J. Gröger) was adopted for this study.

## Results

### Environmental data series

**Fig 2:**
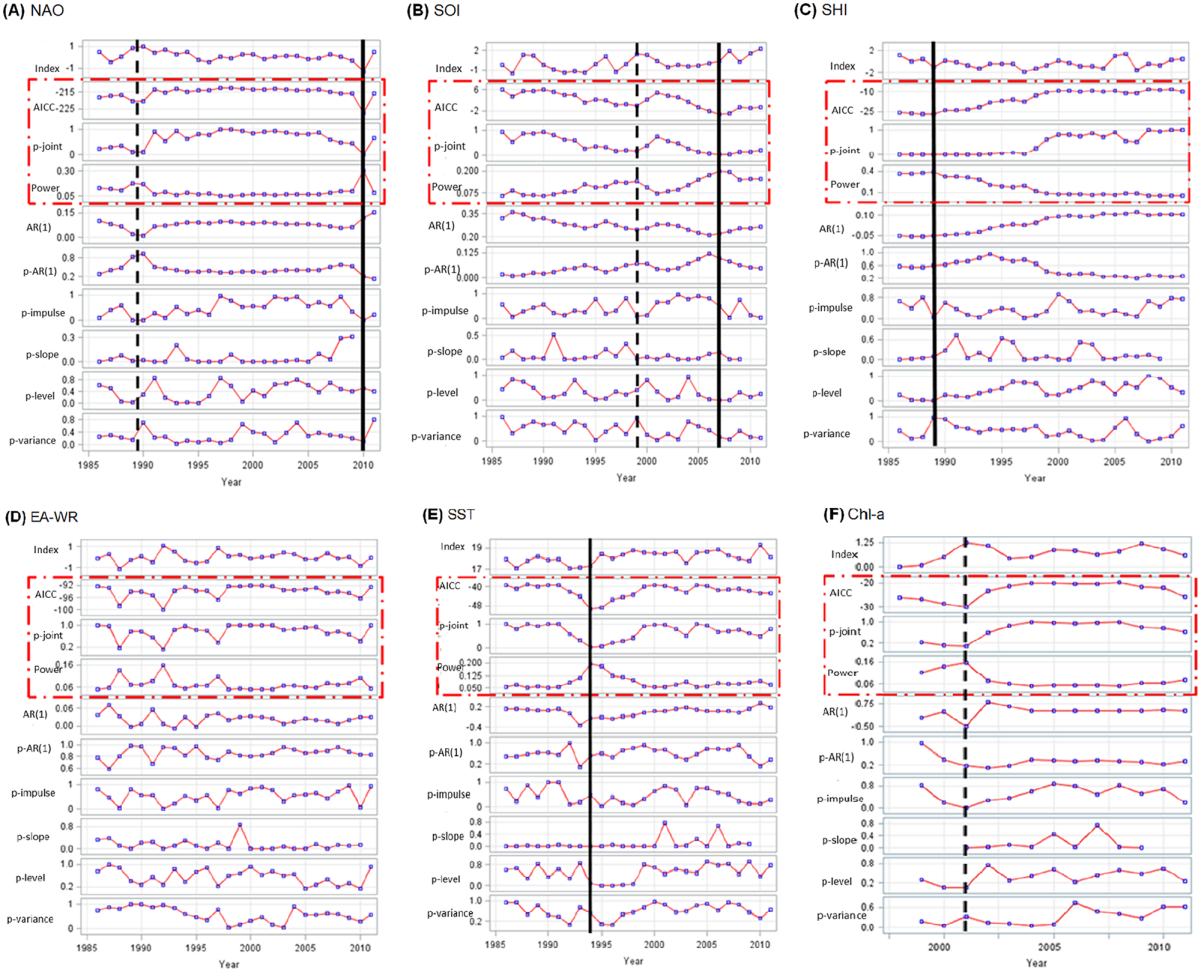
Shiftograms of (A) NAO, (B) SOI, (C) SHI, (D) EA-WR, (E) SST and (F) Chl-a. The black vertical-continuous lines indicate location of strong structural breaks (years of shift occurrence); the black vertical-dashed lines indicate location of prominent(less strong and minor) shift-like signals; the open surrounding rectangle with red-broken lines shows the triple key-statistical indicators for recognizing the breaks (i.e. AICc, p-joint statistics and power).

Fig 2 illustrates the corresponding shiftogram of environmental time series (A-E) and chlorophyll-a time series(F), where the black vertical-dashed lines indicate the years of potential structural breaks.

1. NAO. The center of a major structural break could be identified in year 2010 with a gradual decrease in the level and a sudden increase thereafter: this shock signal is well indicated by the lowest measures of AICc and p-joint and at the same time by the maximum recorded values of power (plots 2, 3 and 4, respectively). P-joint and AICc also indicate that the initial gradual decrease began around 2006. Furthermore, one less strong and rather gradual shift is centered somewhere around (perhaps between) 1989 and 1990.
2. SOI. A rather strong shift could be observed during the period of the study, being centered around 2007. Beside this, the diagram indicates a prominent (but not significant) shift-like signal around 1999.
3. SHI. Local minima of AICc plus local maxima of power indicate a strong shift during the late 1980’s (especially in 1989). Moreover, after 1989 time series shows an obvious gradual upward trend in the AICc and auto-regression.
4. EA-WR. Despite the shiftogram shows repeating bumpy changes along the time series of EA-WR (especially around 1989, 1992, 1997 and 2010), these break-like signals are not confirmed by the AICc, p-joint and power panels.
5. SST. The shiftogram indicates a shift centered at year 1994, with an asymmetric gradual transition period with a shorter initial phase when compared with the outgoing phase. The observed shift has a clear level changing pattern. The main changes in time series characteristics represent a long-transitioning period with rather slow slope changes within a considerable width (8 years). This obvious shift is characterized by an initial gradual decrease in AICc and p-joint began around 1991, reached to the deepest point (local minima) in 1994 and followed by a gradual increase until 2000.
6. Chl-a. During period 1998 to 2011 the chlorophyll shiftogram (Chl-a log-transformed) indicates a rather prominent (but non-significant) break-like signal centered at year 2001. The signal represents a weak indication of an impulse-like (rather flat but not sharp) pattern. This break-like signal is also characterized by an obvious change in auto-regression behavior of the time series. After reaching to the local minima in 2001, the auto-regression initially increased and then reached to a rather plateau-like level by the end of the time series.

### Bentho-pelagic species population components data series

**Fig 3:**
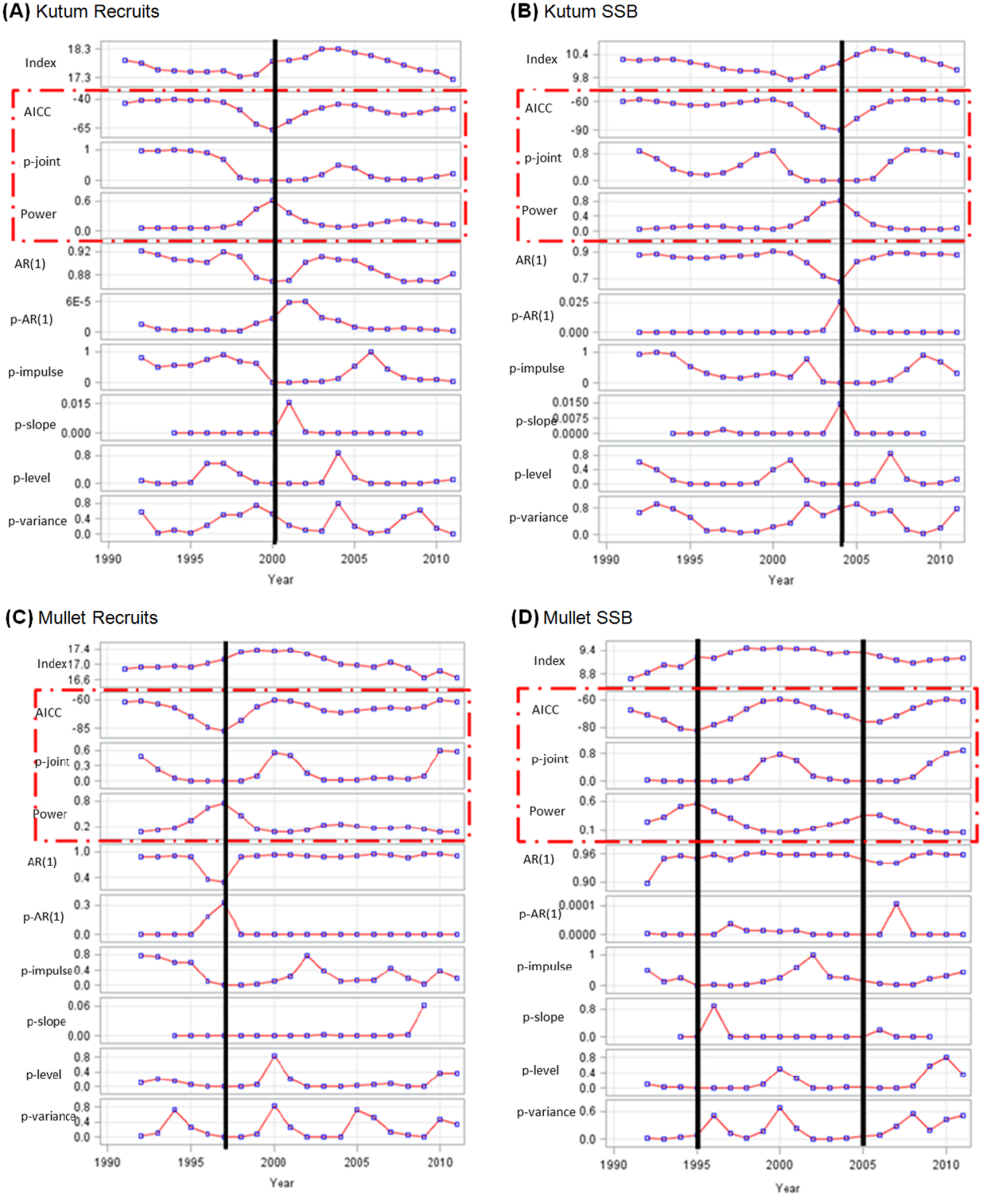
Shiftograms of recruitment and SSB of the Caspian kutum (A and B, respectively) and of golden grey mullet (C and D, respectively). The black vertical-continuous lines indicate location of strong structural breaks (years of shift occurrence); the open surrounding rectangle with red-broken lines shows the triple key-statistical indicators for recognizing the breaks (i.e. AICc, p-joint statistics and power).

Fig 3 illustrates the corresponding shift screening diagrams for the population components (i.e. recruitment Ln (R) and parental stock size Ln (SSB)) related to Caspian kutum (A and B) and golden grey mullet (C and D). The logarithmic-transformed values of population indices were used as inputs for the shift detection algorithm. The results of the shift analysis (Fig 2(A)–2(D)) are described below:

1. Caspian kutum recruitment. Inspection of the shiftogram suggests a strong break in year 2000. This shift is well characterized by the local maxima of the power along with the lowest measures of AICc and p-joint. The changes in the main statistical break features are rather symmetric and short-lasting (around two years before and after shift center). Also inspection of the AICc shows a rather valley-like pattern. Moreover, the auto-regression represents two obvious and separated trends before and after the shift location (downward and upward trends, respectively).
2. Caspian kutum SSB. The shiftogram revealed an evident major structural break around 2003–2004. The major characteristics of this shift are rather similar to those of kutum recruits. However, the change in auto-regression before and after the shift location was a little steeper and more sudden.
3. Golden grey mullet recruitment. The shiftogram suggests a strong shift around 1996–1997 similar to an inflection point. Inspection of AICc shows a rather valley-like pattern initially began about two years before the shift occurrence. The most obvious pattern change in statistical features of the time series is related to the auto-regression behavior: the plateau-like auto-regression pattern appears to be interrupted at years 1996 and 1997 by a reverse valley-type layout.
4. Golden grey mullet SSB. Based on the corresponding key-statistics, the shiftogram indicates two significant shifts, one in 1995 reflecting the steepest (inflection) point of increase, a second less strong one around 2005–2006 reflecting the steepest (inflection) point of decrease. Inspection of the AICc shows a rather symmetric valley-like pattern for both shifts developments. Furthermore, the entire time series shows a very small auto-regression changing pattern.

## Discussion

Reactions of ecosystem components confronting sudden shifts in external driving forces have a quite different character and conceptual nature (qualitative). Such regime changes are related to variations of the mean or variance of a variable and are, thus, not necessarily assessable with conventional correlational statistics and quantitative causal dependency analysis (i.e. the correspondence could not be expressed on metric scale).

The observed structural changes (Fig 2) in global-scale atmospheric forces might potentially act as one of the key-factors triggering the contemporary wide-range alterations in the Caspian ecosystem components, dynamics and functions.

Analyzing data series for detecting the development and timing of shift signals demonstrates the following pattern (summarized in Table 3): regime changes in the atmospheric processes mostly occur from the late 1980’s to the 1990’s, regime shifts in the local temperature conditions (i.e. SST) in the Caspian Sea occur during 1991–2000 and ecological time series exhibit evidences of major shifts during a period between mid-1990’s and mid-2000ies. This might indicate a linkage (a cascade of influences) between external forces and dynamics of ecosystem components. From the shift patterns, it could be hypothesized that external atmospheric processes may be partly involved in (and triggered) manifestation of the regional ecological shifts in the following order: global-scale climate forces imply changes on local environmental processes, which in turn imply biological components dynamics.

**Table 3:** Years of the identified shift patterns and break-like changes of all variables considered in this study.

*Variable*	*1980’s*	*1990’s*	*2000’s*	*2010’s*
***Large-scale climatic indices***				
NAO	1989			2010
SOI		1999	2007	
SHI	1989			
***Local environmental variables***				
SST		1991-1999/2000 (**1994**)		
Chl-a			2001	
***Bentho-pelagic species population’s metrics***				
Caspian Kutum Recruits			2000	
Caspian Kutum SSB			2003–2004	
Golden grey mullet Recruits		1997		
Golden grey mullet SSB		1995	2005–2006	

The NAO and SH show a regime shift in the late 1980’s (near 1989). This regime shift could be considered as the reason for the regime shift in general patterns of physical environment of the Caspian Sea (here SST as the most coherent variable), which starts to occur one or two years later (approximately in 1991). The EA-WR shows no regime shifts and the SOI has two regime shifts in the late 1990’s and around mid-2000ies, i.e., after the regime shift of SST. Therefore, only the SH and the NAO regime shifts seem to be candidates for triggering the SST regime shift.

Authors in [32]found at the inter-annual time scale a relation between local physical conditions of the Caspian Sea (in their case, sea level) and NAO changes. They proposed a mechanism of influence of the NAO on precipitation of the Volga catchment region, but without giving further details. [77](as quoted by [51]) suggested a major influence of the SH on air temperature and moisture. The mechanism proposed was that changes of the SH are related to changes in the atmospheric circulation pattern that favor or block intrusion of cyclonic systems from the Mediterranean region into the Caspian basin. The NAO regime shift being weak (the statistical power is around half of that of SH) in comparison to the SH shift, suggests that most probably the SH shift is the one triggering the SST regime shift.

Our lack of influence of the SO on SST of the Caspian Sea seems in contradiction with [32] and [49]who found a relation between ENSO changes and sea level [32] and the probability of drought in Iran [49]. A reason for this disagreement could be found on the different time scales of those studies: Authors in [32] who found lags of up to 10 years between SO changes and sea level changes, while [49]is related to inter-annual time scales. [49]is also related to meteorological conditions of the southern region of the Caspian Sea (the Caspian Sea local conditions could be more influenced, for instance, through the Volga, which is in the north, which is surely the case for sea level). We only found, in agreement with the notions of [32] and [49]an evidence of a potential relation between the SO and Chl-a: There is a relatively weak regime shift of SO in 1999, which is followed by a rather break-like signal in Chl-a data series in 2001.

It seems that the regime shift in the thermal condition (as one of the most striking abiotic factor) could provide a basic explanation for understanding of potential climate-induced shifts in bentho-pelagic subsystem in the Caspian Sea. All the prominent shift events in biological system components (a period between mid-1990’s and mid-2000ies; Fig 3 and Table 3) basically followed the long transitioning shift signal in local environmental processes (SST). Thus, the local temperature regime change (during 1991–2000) appears to be one of the driving forces behind occurrence of shift signals in both bentho-pelagic fish population proxies (both recruits and parental stocks) in the Caspian Sea.

Qualitative correspondence and comparable timing of biological responses of bentho-pelagic stocks (expressed by the population proxies) to environmental events (particularly here the regime change of SST) is one of the supporting evidences that overall Caspian ecosystem structures and functioning might have–at least partially–been impacted by climate-induced shifts. Furthermore, the concurrent shift-like signal in primary production (represented in Chl-a data series, covering period 1998–2011) could be assumed as a potential sign of coupled (interlinked) physical-ecological shifts influencing trophic structures/interactions and cascading intermediate food-web processes.

The explorative findings from the qualitative serial shift analyses in this study tend to correspond well with a series of parallel events and recent substantial ecosystem-wide changes in the southern Caspian Sea (i.e. overlap in temporal zone). In this context, a comprehensive review by [39] suggested several empirical evidences of significant biotic alterations in the southern Caspian environment (particularly between mid-1990’s and mid-2000’s) including:

- Major change in primary and secondary trophic production of the ecosystem expressed by significant variations in demographical-phenological traits of local planktonic communities (both phyto- and zooplanktons structures) involving species diversity, abundance, biomass, size composition and seasonal blooming events.
- Appearance, increase in biomass, spatial distribution and outburst of the invasive comb jellyfish (*Mnemiopsis leidyi*) population.
- Structural changes in macrobenthic fauna including biodiversity, biomass, abundance and partial replacement of zoobenthic species.
- Drastic variations in local commercial fisheries especially decrease in catch and collapse of pelagic Kilka species.

A rough simultaneity of the above-mentioned structural changes in various ecosystem compartments, biodiversity and trophic structures and interactions in the southern Caspian Sea, as well as the identified ecological shift signals appear to be (at least in part) driven by physical processes caused by integration of exogenously-forced climatic feedback mechanisms. Taken together, these evidences may support that the Caspian Sea has likely encountered regime shift by climate forcing under on-going environmental changes (such as global marine ecosystems [21–27]). However, a robust statement on occurrence of an entire regime shift in the Caspian Sea requires a continuation of investigations on many other biotic and abiotic variables to provide further supporting evidences.
